# Effects of Decabromodiphenyl Ether (BDE-209) on Inter-Specific Competition between Two Species of Marine Bloom-Forming Microalgae

**DOI:** 10.1371/journal.pone.0056084

**Published:** 2013-03-21

**Authors:** Xinxin Zhang, Xuexi Tang, Bin Zhou, You Wang

**Affiliations:** Department of Marine Ecology, College of Marine Life Sciences, Ocean University of China, Qingdao, China; University of Illinois at Chicago, United States of America

## Abstract

Decabromodiphenyl ether (BDE-209), a new kind of persistent organic pollutants, was selected to investigate its influence on population growth and inter-specific competition between two species of marine bloom-forming microalgae, *Heterosigma akashiwo* and *Karenia mikimotoi*. (1)BDE-209 showed acute toxic effects on both microalgae and *H. akashiwo* was more sensitive from view of 96 h-EC_50_ and the ultrastructure variation. (2)The microalgal population growth patterns in mono-culture were density-dependent and the growth of both species in the normal co-culture was significantly depressed by competition (*P*<0.05) with different initial biomass ratios. BDE-209 exposure significantly changed the growth. (3) Lotka-Volterra competition model was used to simulate the interaction between the microalgae. BDE-209 exposure broke the competitive balance to make competition gradually shift in favor of *H. akashiwo*. Results suggested BDE-209 did have toxic effects on either microalgal growth or the inter-specific competition, which was quite different from previous reports. Further exploration of the mechanism is needed.

## Introduction

Polybrominated diphenyl ethers (PBDEs), the new kind of persistent organic pollutants (POPs) which are structurally similar with Polychlorinated biphenyls (PCBs), are now widely used as additive flame retardants in household and commercial products, especially in electronic ones [1], [2]. However, PBDEs could be released into the environment via the manufacturing process and handling, disposal or recycling of the treated products, which would contaminant the environment [3], [4], [5], [6]. It is not only the residential areas but also the polar zone and deep sea are reported the presence of these substances [7], [8]. Moreover, PBDEs have the distinct characteristics of bio-accumulation and bio-transformation, and both the terrestrial and the aquatic organisms have been found the presence of PBDEs [9], [10], [11]. PBDEs have become global pollutants and have aroused the worldwide attention.

The endocrine toxic effects of PBDEs have been documented. For instance, the oral route of deca-BDE affected the thyroid hormone homeostasis of weanling rats [12], and the possible mechanism was that PBDEs elicited toxicity by binding to the transport proteins for thyroid hormones and thus altering thyroid homeostasis [13]. They also could antagonize with female hormone to disrupt the processes of development and reproduction. Low-dose BDE-99 exposure during development caused hyperactivity in the offspring and permanently impaired spermatogenesis by the mean of reduced sperm and spermatid [14]. Another study found that PBDEs exposure increased some metabolizing enzymes of rats which indicated they had the potentially reproductive toxicity [15]. Moreover, PBDEs could disturb the animals' motion, behavior and memory by interfering with the neural system [16].

However, few literatures have focused on the toxic effects of PBDEs on algae, and the obtained results were contradictive. For example, the risk evaluation of PBDEs by the European Commission showed that Deca-BDEs exerted very low toxicity in acute tests for algae with the concentrations being up to the water solubility limit [17]. Moreover, no effects were also expected to occur in the short term tests at concentrations up to the solubility limit of Octa-BDEs by the analogy with another highly brominated diphenyl ether (decabromodiphenyl ether) [18]. In contrast, Källqvist et al. found that BDE-47 caused obvious growth inhibition in the marine diatom *Skeletonema costatum*, and the no observed effect concentration (NOEC) was 6.6 mg/L [19]. BDE-47 had also been found to depress the growth of four species of marine microalgae and affect the activities of antioxidant enzymes [20]. Our recent studies have found that the low brominated congeners (BDE-47, penta-BDEs) damaged the microalgal growth [21]. Nevertheless, the data about higher brominated ones (e.g. BDE-209) were scarce.

Interspecific competition plays a desicive role in the diversity and stability of microalgal community [22]. Microalgal competition is affected by both abiotic and biotic factors. For example, fluctuations in light and nutrient levels could result in the switching of dominance; the opportunist species that were most adaptive to these environmental changes would have a competitive advantage [23]. As to biotic factors, such factors as life history strategies, nutrient-absorbing strategies, migration, and allelopathy could affect inter-specific competition in the community [24], [25]. Interactions between different species of microalgae had been studied mathematically by many authors [26], [27], [28], [29], [30]. Solé et al. [31] estimated the interactions between two microalgal species based on a model proposed by Chattopadhyay [32], and this study suggested a functional form suitable for quantifying the strength of interaction between algae. Wang et al. [28] reported the interactions between two species of bloom dinoflagellates, *Alexandrum tamarense* and *Prorocentrum donghaiense*, in bi-culture and estimated the strength of their interaction by a mathematically model proposed by Uchida et al. [30].

Deca-substituted BDE-209 (3,3′,4,4′,5,5′,6,6′-decabromodiphenyl ether) is the primary component of commercial deca-BDE (typically≥97%), which constitute approximately 80% of the world market demand for PBDEs [33], [34]. Currently, BDE-209 has been found at different levels in abiotic and biotic compartments [3], [10], [35]. The worldwide presence of BDE-209 indicates that the emphasis should be focus on ascertaining its toxicity. Thus, the discharge of e-waste with a large amount of BDE-209 will aggravate the environmental burden. *Heterosigma akashiwo* and *Karenia mikimotoi* are marine bloom-forming microalgae that have caused many red tides in recent years [36], [37]; more information is needed on the population growth and inter-specific competition of these species.

We therefore chose BDE-209 as the target pollutant, and two main bloom-forming microalgae as the tested organisms. The purpose of the present study is to illuminate the acute and chronic toxic effects of BDE-209 on the growth and inter-specific competition of microalgae under controlled laboratory conditions. The possible effective mechanism is discussed, and the inter-specific competition is quantified using the Lotka-Volterra two species competition model. Our goals are to provide some theoretical help in microalgal competition and to develop a better understanding of BDE-209 toxicity for the purpose of the reasonable use and disposal of electronics.

## Materials and Methods

### 1. The prepared BDE-209 stock

2,2′,3,3′,4,4′,5,5′,6,6′-decabromodiphenyl ether (BDE-209, C_12_Br_10_O), a kind of white powder, was used in the present study. It was provided by Dr. Ehrenstorfer Laboratories (purity 99.5%) and purchased from Quandao Company (Shanghai, China) Dimethylsulfoxide (DMSO) was used as the organic solvent (HPLC grade, AMRESCO, USA). BDE-209 was dissolved in DMSO to prepare the stock solution and was then diluted to the required concentrations according to a preliminary experiment.

The actual concentrations of BDE-209 in the culture medium were measured by high performance liquid chromatography (HPLC). The liquid chromatograph was Hitachi L-2000. The mobile phase consists of buffer solution A (Na_2_HPO_4_, 3.39 g L^−1^; KH_2_PO_4_, 3.35 g L^−1^) and solution B (HPLC grade methanol). The gradient elution program was as follows: 0–15 min (95% B, 0.1 mL/min), 15 min (100% B). The temperature of the column oven was set at 25°C, and the flow rate was 1.0 ml min^−1^. The detective wavelength was 340 nm. 20 µl of the test solution was injected into the HPLC system by an auto injector. A standard curve was generated directly by reverse phase HPLC between the stock concentration of BDE-209 and its waterborne concentration [38]. Then a regression relation between the concentrations of BDE-209 (c) and their area of integral (A) by HPLC was obtained as A = 10434.84c-3633.73 (R^2^>0.9999). The tested concentrations used in the present study were estimated according to the standard curve, and then was found to be less than 0.00426 mg L^−1^ in all levels of BDE-209 during the whole study.

Quality assurance/Quality control. All the glass instruments were used after high-temperature roasting with the exception of the plastics. The BDE-209 solution was kept in dark place during the test to avoid photolysis. The credibility of the external standard method was valuated as follows and all determinations were performed in the same conditions: the five samples were consecutively injected from the same preparation (*RSD* = 0.8%). And then 20 µl of test solution was injected to the system every two hours for eight hours and it was found that the results had no marked change (*RSD* = 1.2%). There were five identical samples used for duplicate test (*RSD* = 1.7%). The mean recovery rate was about 98.5% (*n* = 5). The used HPLC system accuracy and precision were examined on the basis of the recovery rates and RSD. It was stated that system accuracy and precision were satisfactory according to the executed statistical determination.

### 2. The microalgae culture

*Heterosigma akashiwo* (Raphidophyta) and *Karenia mikimotoi* (Dinophyta) were kindly provided by the Algal Center of the Ocean University of China. The algae were grown in closed Erlenmeyer flasks with modified f/2 media [39] at 22±1°C 80 µmol photon m^−2^ s^−1^ with a 12 h light∶dark cycle in illuminating incubators. The initial pH and salinity of the culture medium were adjusted to 8.0±0.02 and 30, respectively. During the whole experiment, light intensity and temperature were gauged by the JD-3 luxmeter (Shanghai Jiading Instrument, China) and ordinary mercurial thermometer at regular intervals, respectively. The levels of nitrate and phosphate were estimated colorimetrically by the zinc-cadmium [40] and phosphomolybdenum blue reagents [41] at the end of the experiment. Flasks containing the microalgae were shaken manually twice at set times of one day. The microalgae were cultivated to the exponential growth phase for use. The total experimental volume was 250 ml, and a 1-mL sample was collected daily and preserved in Lugol's solution to estimate the microalgal growth by directly counting cell numbers using a hemocytometer under an optical microscope (Motic SFC-18, Motic China Co. Ltd, Xiamen, China). *H. akashiwo* is easily distinguished from *K. mikimotoi* by size, shape and swimming pattern.

### 3. The concentrations of BDE-209 in acute and subchronic toxicity test

No observed effect concentration (NOEC) of DMSO was 0.75% (v/v) for *H. akashiwo* and *K. mikimotoi* in the preliminary experiment. The BDE-209 acute toxicity test was performed according to Swedish standard procedures [42]. The concentrations of BDE-209 within the safety range of DMSO (≤0.3%, v/v) were set to the following values with a logarithmic equal-interval of log2 (Table 1).

**Table 1:** Concentrations of BDE-209 were set in the acute toxicity test for two species of microalgae.

	Concentrations of BDE-209 (mg L^−1^)						
*H. akashiwo*	0	5	10	20	40	80	-
*K. mikimotoi*	0	5	10	20	40	80	160

The 96 h-EC_50_, the interpolated concentration at which algal population growth would be inhibited by 50% over 96 h, was estimated using straight-line graphical interpolation [43]. The initial cell density was set at 1×10^4^ cells mL^−1^ for both algae to exclude the influence of initial cellular densities on the determination of the EC_50_ [44]. The 96 h-EC_50_ values of *H. akashiwo* and *K. mikimotoi* were used as the basis for further sub-lethal toxic effects in the co-culture. The relative growth rate was defined by the following formula:
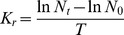
(1)Where *K*_r_ denotes the relative growth rates, *N_0_* and *N_t_* are the population densities at times 0 and *t*, and *T* is the time interval.

In the mono-cultures, the initial cell densities of *H. akashiwo* and *K. mikimotoi* were showed in the Table 2, and those data can be used as the respective controls for co-culture. Interactions between phytoplankton are expected to be size-dependent due to surface/volume considerations [45]. Therefore, the initial cell densities for *H. akashiwo* (abbreviated as H in the experiment) and *K. mikimotoi* (abbreviated as K in the experiment) in the bi-algal culture were adjusted according to their respective cell volumes so that the size/density ratios would be 4∶1, 1∶1 or 1∶4.

**Table 2:** The initial cell densities of microalgae were set in the aquatic test, monoculture and co-culture (×10^4^ cells mL^−1^).

	Aqutic test	Mono-culture	Co-culture(H∶K = 4∶1)	Co-culture(H∶K = 1∶1)	Co-culture(H∶K = 1∶4)
*H. akashiwo*	1	0.5 0.8 3.2	3.2	0.8	0.5
*K. mikimotoi*	1	0.2 0.5	0.2	0.2	0.5

Then the stock solution of BDE-209 was added into the microalgal mono- and co- culture medium so that two final concentrations were obtained based on the calculated 96 h-EC_50_ values; the high concentration treatment was 18 mg L^−1^, and the low concentration treatment was 1.8 mg L^−1^. The initial biomass ratios in the bi-algal cultures were the same values as the ones described in co-culture without BDE-209. Two controls were used in this experiment: a blank control of microalgae grown in f/2 medium without BDE-209 addition, and a negative control of microalgae grown in DMSO (0.3%, v/v). Both the mono-cultures and co-cultures lasted for 18 days.

### 4. The preparation of samples for transmission electron microscope (H-7000TEM, Japan)

The microalgal suspensions from different treated groups in the acute toxicity test were centrifuged at a rotating speed of 3500×*g* at 4°C for 15 min and as much of the supernatant as possible was then removed. Subsequently, the microalgae cells were immersed in a fixative liquid containing 5% glutaraldehyde in 0.05 mol L^−1^ potassium phosphate buffers (pH 7.4). The treated samples were kept at 4°C overnight in the fixative liquid and washed 3 times using the fixative liquid every 30 min the next day. This preparation was carried out in the absence of sunlight. Slices were prepared according to Zhu (1983) [46].

### 5. Statistical analysis

The experimental data were log-transformed, and a logistic growth curve was fitted with a logistic growth model. The following logistic formula was applied:
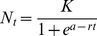
(2)Where *N_t_* is the cell density at time *t* (10^4^ cells mL^−1^), *K* is the carrying capacity of the population (10^4^ cells mL^−1^), *t* is the time interval (d), *r* is the maximal specific growth rate (d^−1^), and *a* is a constant related to the initial cell density of algae (*N_0_*) (*a* = ln((*K-N_0_*)/*N_0_*)). The formula was applied for the growth phase before the maximal cell density was reached. The maximal population growth rate (*r*), the constant (*a*), and the carrying capacity (*K*) were estimated using nonlinear regression in SPSS version 16.0 by applying the equation (2). Mean values and standard deviations were calculated from the different replicates from each treatment (*n* = 3), and figures were generated using Sigmaplot 10.0. Differences between treated groups and controls were analyzed by one-way ANOVA in SPSS version 16.0 with significance set at *P*<0.05.

## Results

### 1. Acute toxic effect of BDE-209 on the two species of microalgae

**Figure 1:**
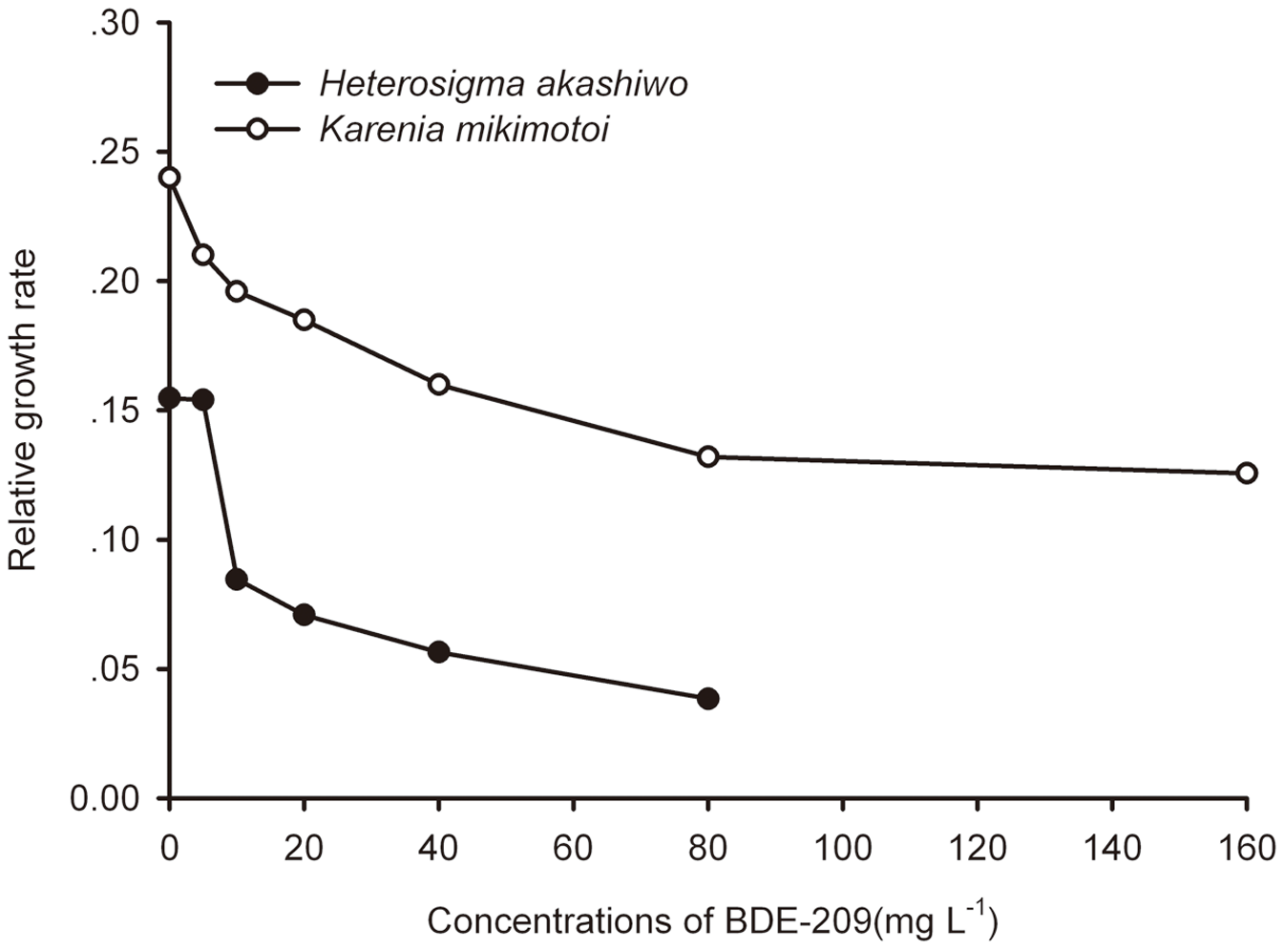
Acute toxic effects of BDE-209 on the relative growth rates of microalgae at 96 h.

In the acute toxicity tests, the relative growth rates of *H. akashiwo* and *K. mikimotoi* decreased dramatically with increasing concentrations of BDE-209. The 96 h-EC_50_ values were 22.58 mg L^−1^ for *H. akashiwo* and 120.8 mg L^−1^ for *K. mikimotoi* (Figure 1). Any effect of DMSO on the microalgae could be ignored because of the negative control.

### 2. Effect of BDE-209 on ultrastructure of *H. akashiwo* and *K. mikimotoi*

**Figure 2:**
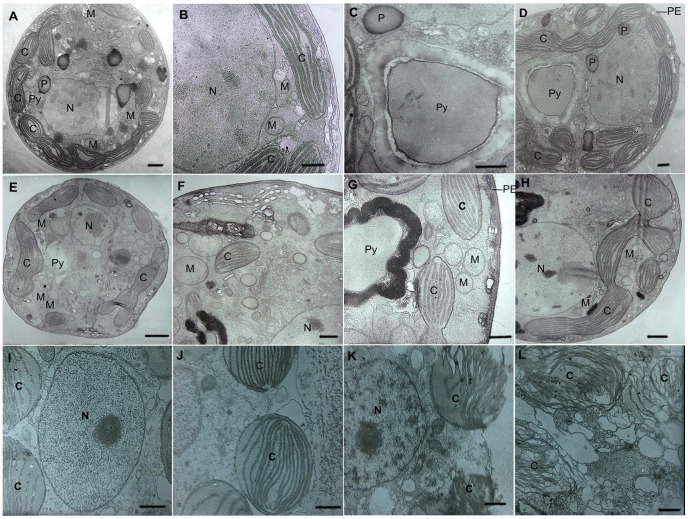
The ultrastructure of microalgae cells in controlled groups and the BDE-209 treated groups. (**A**), (**B**), (**C**) and (**D**) *K. mikimotoi* cell was oval with complete cytomembrane, chloroplast, mitochondria, nucleus and pyrenoid in controlled groups; (**E**), (**F**), (**G**) and (**H**) *K. mikimotoi* cell was out of the shape while the cell membrane was thickened and organelle were broken in treated groups; (**I**) and (**J**) The nucleus and chloroplasts were intact for *H. akashiwo* in controlled groups; (**K**)and (**L**) The nucleus underwent a slight transformation with some compact spots of dense chromatin and the chloroplasts were broken, and the structure of lamellae was indistinct for *H. akashiwo* in treated groups. PE, P, C, M and N denoted cytomembrane, pyrenoid, chloroplast, mitochondria, and nucleus, respectively. The scale of 1 µm was indicated at the bottom of every figure.

In the control group, *K. mikimotoi* cells were oval in shape with clear outer membranes (Figure 2A). The chloroplast of the cell was intact, and inside the chloroplast the thylakoid was arranged neatly and the grana were folded together (Figure 2B). The cristae of the mitochondria were visible and the Golgi body was tightly arranged. The cytoplasm, proteasomes and pyrenoids were distinct (Figure 2C). The nucleus, including an uninjured nuclear membrane and equally distributed nucleoplasm, was in good condition (Figure 2D). However, the subcellular structure was destroyed upon exposure to BDE-209. The cell swelled, the cellular shape changed to round, and the cell membrane was thickened. The chloroplast became distended, and the number of chloroplasts decreased. The lamellae of the thylakoid were fractured, partially obliterated and even broken down such that it became difficult to distinguish one from the other. The pyrenoids were atrophic and the previously transparent starch sheath turned black. The number of mitochondria increased and the segments of cristae were broken. Furthermore, chromatin formed many dense zones. More vacuoles with irregular shapes and blank spaces were observed (or occurred) in the cytoplasm (Figure 2E,2F,2G,2H).

For *H. akashiwo*, it was difficult to observe the whole cell in both control groups and treated groups, but differences in the ultrastructure of chloroplasts and nuclei were easy to distinguish. In the control group, the nucleus was intact and had a well-distributed nucleoplasm and a clearly visible nucleolus (Figure 2I). Additionally, most of the chloroplasts were intact and the structure of lamellae was obvious (Figure 2J). When exposed to BDE-209, however, the nucleus underwent a slight transformation in that there were some compact spots of dense chromatin (Figure 2K). The chloroplasts were broken, and the structure of lamellae was indistinct (Figure 2L).

### 3. Microalgal growth in mono-culture with different initial cell desities

**Figure 3:**
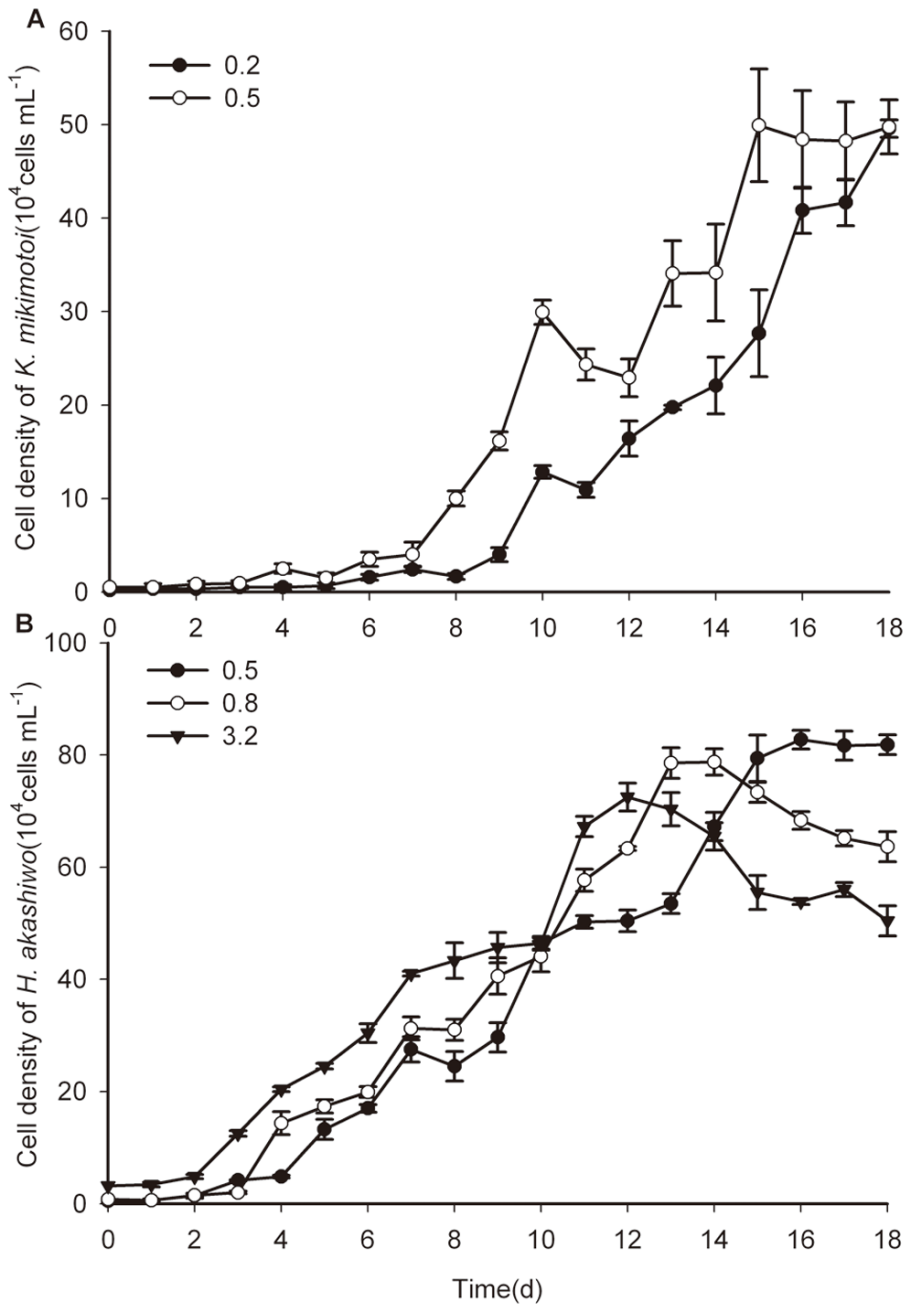
The growth curves for microalgae at different initial cell densities. (**A**) The population growth of *K. mikimotoi* with the initial cell densities of 0.2×10^4^ cells mL^−1^ and 0.5×10^4^ cells mL^−1^ (**B**) The population growth of *H. akashiwo* with the initial cell densities of 0.5×10^4^ cells mL^−1^, 0.8×10^4^ cells mL^−1^ and 3.2×10^4^ cells mL^−1^. Data were expressed as means ± SE (*n* = 3).

The growth curve of *K. mikimotoi* and *H. akashiwo* in the mono-culture is shown in Figure 3. In the first 7 days, both of the growth with two initial cell densities was slow for *K. mikimotoi*. Subsequently, cells in the 0.5×10^4^ cells mL^−1^ group grew significantly faster than those in the 0.2×10^4^ cells mL^−1^ group (*P*<0.05). It was estimated that the time to enter the exponential growth phase (*T_EP_*), stationary growth phase (*T_SP_*) and the time to reach the inflection point (*T_P_*) were shortened when the cell densities increased. An obvious difference was observed between their individual carrying capability (*K*, 0.01<*P*<0.05). At initial cell densities of 0.5×10^4^, 0.8×10^4^ and 3.2×10^4^ cells mL^−1^, *H. akashiwo* grew slowly in the first 2 days and later entered the exponential phase on the 2nd, 3rd and 4th days, respectively. *T_SP_* was shortened and the value of *K* decreased significantly (*P*<0.05) as the initial cell density increased.

### 4. Inter-specific competition between *H. akashiwo* and *K. mikimotoi* in co-culture with and without BDE-209 exposure

#### 4.1. Inter-specific competition without BDE-209 exposure

**Figure 4:**
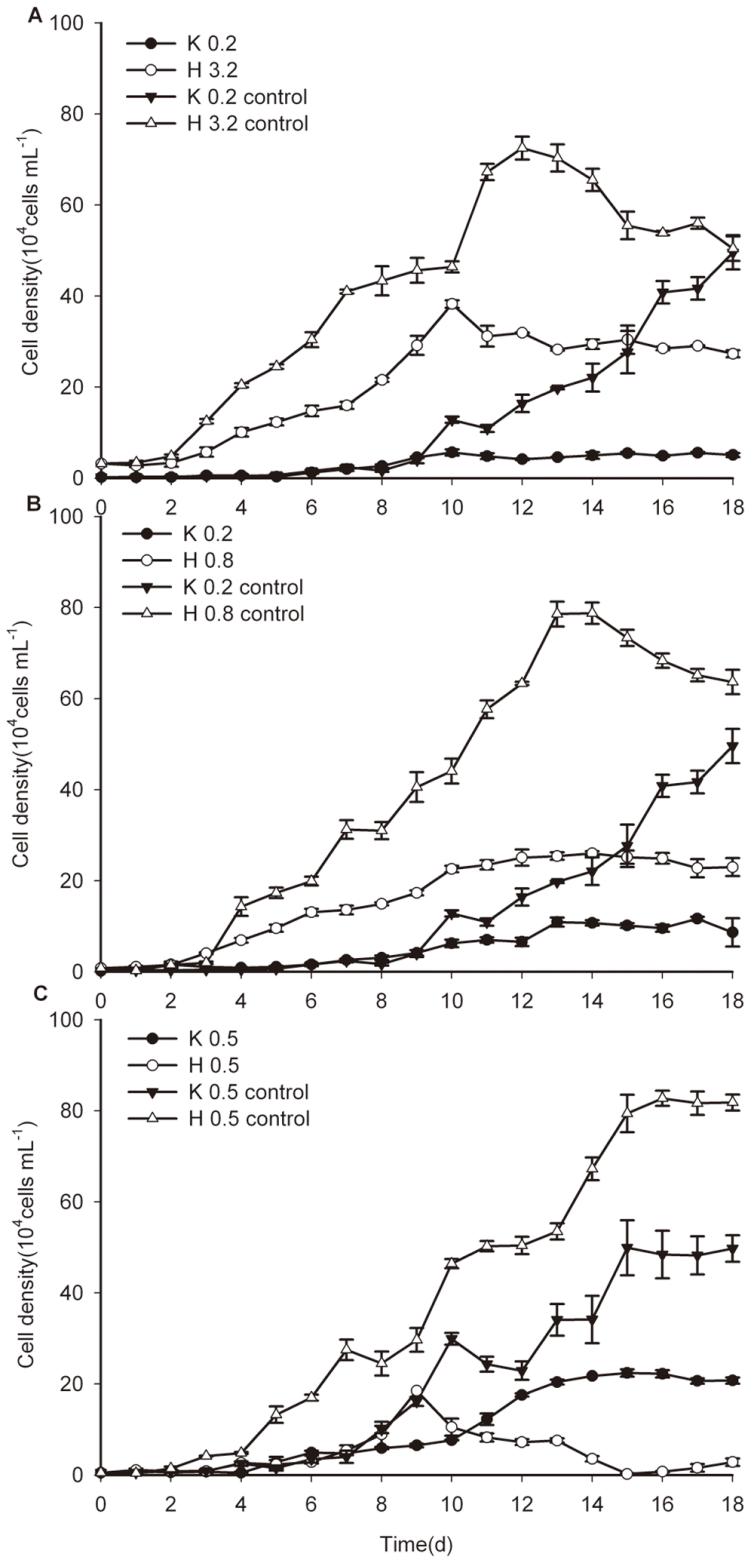
The growth curves of microalgae in the co-culture without BDE-209 at the initial biomass ratios. (**A**)The initial biomass ratio was set at H∶K = 4∶1. (**B**) The initial biomass ratio was set at 1∶1. (**C**) The initial biomass ratio was set at H∶K = 1∶4. Data were expressed as means ± SE (*n* = 3).

With regard to inter-specific competition in the absence of BDE-209, the growth of both *H. akashiwo* and *K. mikimotoi* was depressed compared with their respective control groups and that of *K. mikimotoi* was relatively slower when the initial biomass ratio was equal to H∶K = 4∶1. For *H. akashiwo*, the population growth was obviously suppressed relative to the control (*P*<0.01), and the *K* value was about 50.23% of that in the mono-culture. The growth regression equation for *H. akashiwo* in the co-culture was estimated as *N* = 30.6690/(1+e^3.4152-0.5909t^) (*R* ^2^ = 0.9265). For *K. mikimotoi*, the growth was significantly inhibited, and the *K* value was only 6.9% of that in the control group (*P*<0.05). No visible exponential phase was observed throughout the experiment (Figure 4).

A similar result was obtained in the co-culture when the initial inoculation ratio changed to 1∶1. *H. akashiwo* grew faster and entered the exponential phase more rapidly than *K. mikimotoi* (Figure 4). The values of both *T_SP_* and *T_P_* decreased while the maximum growth rate (*r*) increased for two species of microalgae. As to *H. akashiwo*, the *K* value declined to 27.84% of that in the control group (*P*<0.01) and the growth regression equation of was estimated to be *N* = 25.0804/(1+e^3.0727-0.4810t^) (*R* ^2^ = 0.9776). For *K. mikimotoi*, the *K* value was only 20.26% of that in the control, and a significant difference was observed between that in the co-culture and in the control (*P*<0.01). The growth regression equation for *K. mikimotoi* was estimated to be *N* = 11.4439/(1+e^4.0283-0.3520t^) (*R* ^2^ = 0.9673).

When the initial biomass ratio was H∶K = 1∶4, significant growth suppression occurred for both *H. akashiwo* and *K. mikimotoi* in the co-culture relative to their respective control groups (*P*<0.01). Additionally, the growth inhibition of *H. akashiwo* seemed more severe than that of *K. mikimotoi* (Figure 4) and could not be simulated by the logistic growth model equation (*R* ^2^ = 0.2395). It seemed that *K. mikimotoi* had the competitive advantage. The growth regression equation of *K. mikimotoi* was estimated to be *N* = 23.0079/(1+e^5.3541-0.5203t^) (*R* ^2^ = 0.9717).

#### 4.2. Inter-specific competition with BDE-209 exposure

**Figure 5:**
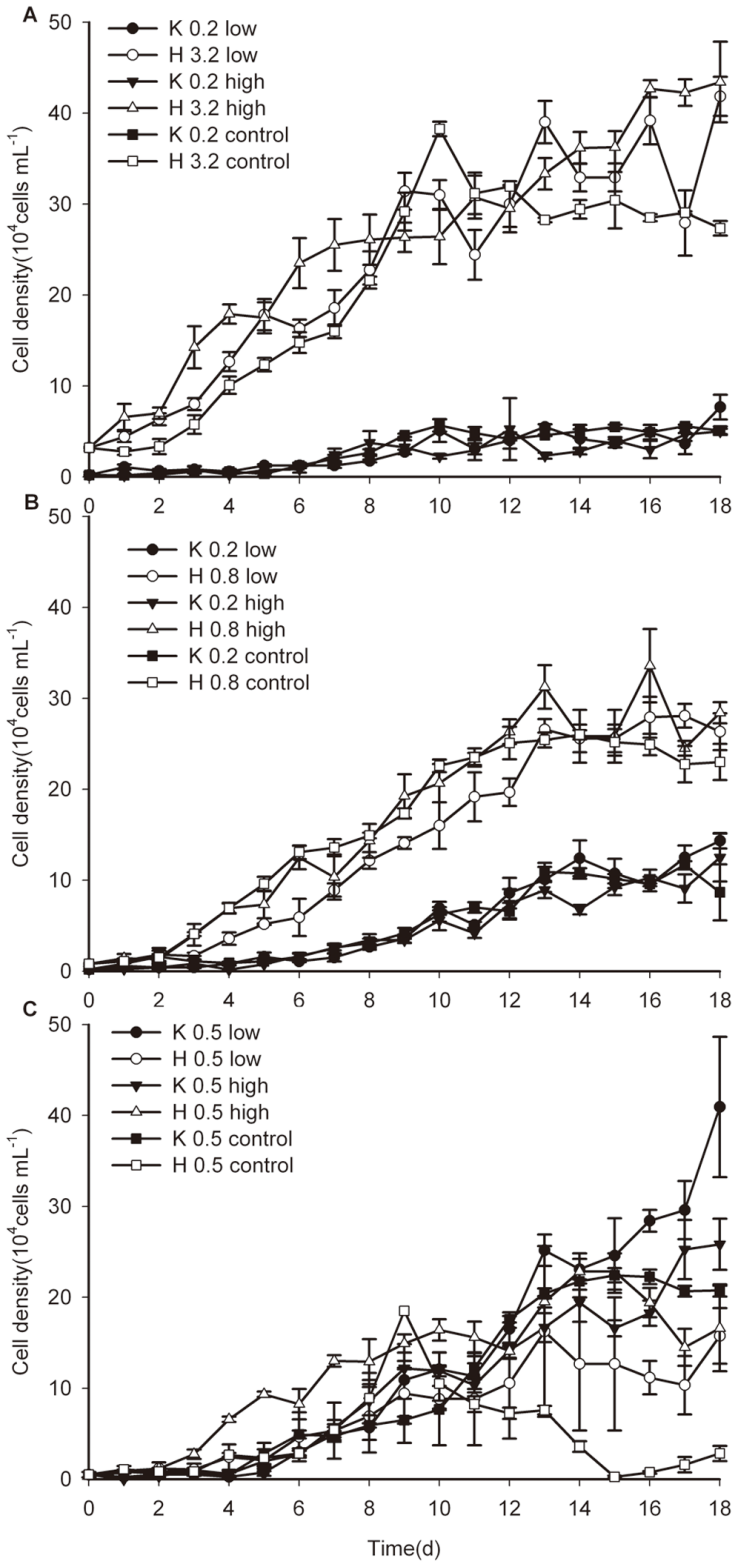
The growth curves of microalgae in the co-culture with BDE-209 at the initial biomass ratios. (**A**) The initial biomass ratio was set at H∶K = 4∶1 with the low toxic (1.8 mg L^−1^) or high toxic (18 mg L^−1^) BDE-209 exposure. (**B**) The initial biomass ratio was set at H∶K = 1∶1 with the low toxic (1.8 mg L^−1^) or high toxic (18 mg L^−1^) BDE-209 exposure. (**C**) The initial biomass ratio was set at H∶K = 1∶4 with the low toxic (1.8 mg L^−1^) or high toxic (18 mg L^−1^) BDE-209 exposure. Data were expressed as means ± SE (*n* = 3).

The two microalgal species behaved differently when the initial biomass ratio was H∶K = 4∶1 under low (1.8 mg L^−1^) and high (18 mg L^−1^) BDE-209 exposure. Throughout the experiment, the population growth of *K. mikimotoi* was strongly suppressed, and no obvious exponential or stationary phases were observed in the three *K. mikimotoi* groups. In the case of *H. akashiwo*, several parameters differed among the control, low toxicity, and high toxicity groups. For instance, the *K* value increased from 30.6690×10^4^ cells mL^−1^ (control group) to 36.5252×10^4^ cells mL^−1^ (low toxicity group, 0.01<*P*<0.05) and 46.0042×10^4^ cells mL^−1^ (high toxicity group, 0.01<*P*<0.05) and so did the *T_p_* value. Conversely, the *r* value decreased. The growth regression equation was estimated to be *N* = 36.5252/(1+e^2.1613-0.3494t^) (*R* ^2^ = 0.9107) in the low toxicity group and *N* = 46.0042/(1+e^1.6176-0.2164t^) (*R* ^2^ = 0.9503) in the high toxicity group. However, the *K* value of *H. akashiwo* was not significantly different at the *P*<0.05 level between the low and high toxicity groups. Meanwhile, *H. akashiwo* appeared to take the competitive advantage in both treated groups and the control group (Figure 5A).

The result of the inter-specific competition changed when the initial biomass proportion was altered to H∶K = 1∶1. For *K. mikimotoi*, the significance of *K* value was observed (0.01<*P*<0.05) between the low toxicity and control groups, but the K value of the high toxicity group was not significantly different from the control at the *P*<0.05 level. The growth regression equation of the low toxicity group was estimated to be *N* = 13.2915/(1+e^5.0395-0.4598t^) (*R* ^2^ = 0.9520) while the simulated growth regression equation of the high toxicity group was *N* = 12.4799/(1+e^3.7775-0.3233t^) (*R* ^2^ = 0.9505). For *H. akashiwo*, no obvious difference was observed between the treated and control groups, and the *K* and *r* values in the low and high toxicity groups were not significantly different from the corresponding control groups at the *P*<0.05 level. The growth regression equation was estimated to be *N* = 28.7673/(1+e^3.6140-0.3964t^) (*R* ^2^ = 0.9897) in the low toxicity group and *N* = 29.3092/(1+e^3.2286-0.4247t^) (*R* ^2^ = 0.9556) in the high toxicity group (Figure 5B). Moreover, a paired t-test showed that both the microalgal *K* values were not significantly different between the low and high toxicity groups at the *P*<0.05 level.

When the initial biomass ratio was changed to H∶K = 1∶4, the competitive result could not reflect with simply relying on Figure 5C. In the low toxicity group, the *K* value had the difference with that of the control group (0.01<*P*<0.05) for *K. mikimotoi* and had no significance (*P*>0.05) for *H. akashiwo*. In the high toxicity group, the *K* value was 31.4937×10^4^ cells mL^−1^ (*P*>0.05) for *K. mikimotoi* and was 18.6748×10^4^ cells mL^−1^ (*P*<0.01) for *H. akashiwo*, respectively. The growth regression equation of *K. mikimotoi* was fitted using the logistic growth equation and was estimated to be N = 45.2492/(1+e^4.5622-0.3303t^) (*R* ^2^ = 0.9669) in the low toxicity group and N = 31.4937/(1+e^3.5917-0.2770t^) (*R* ^2^ = 0.9578) in the high toxicity group. The growth regression equation of *H. akashiwo* was estimated to be N = 13.2452/(1+e^3.4490-0.4407t^) (*R* ^2^ = 0.9024) and N = 18.6748/(1+e^2.8435-0.4814t^) (*R* ^2^ = 0.9006) in the low and high toxicity groups, respectively. Furthermore, the *K* values of *H. akashiwo* and *K. mikimotoi* were not significantly different at the *P*<0.05 level between the low and high toxicity groups, respectively.

## Discussion

The results showed that BDE-209 had toxic effects on *H. akashiwo* and *K. mikimotoi*. The relative growth rates of both species decreased steadily with increasing concentrations of BDE-209, additionally *H. akashiwo* seemed to be more sensitive. This result was quite different from some previous reports, which demonstrated that PBDEs, especially higher brominated congeners such as deca-BDEs, had little toxic effect on microalgae [17], [47]. Another previous report [21] showed that some higher brominated congeners like BDE-209 were less toxic than the lower brominated congeners (e.g., BDE-47). It was suggested that the less toxicity of BDE-209 was likely due to its high molecular weight or size, which may result in its inefficient uptake by organisms. Therefore, it had been claimed that BDE-209 had no ecotoxicological importance because of this assumed negligible bioavailability [48]. However, this issue has recently been of interest because deca-BDEs have been detected at high concentrations in abiotic environmental samples [49] as the major commercial PBDEs product [50]. Moreover, analyses of organisms from both the Atlantic Ocean and the more polluted Baltic Sea have shown the presence of BDE-209 and have inferred its toxicity not to be negligible [51].

On the other hand, some malformations of ultrastructure, exhibited in microalgae upon exposure to BDE-209, have been observed under many stress conditions including metal toxicity [52], acidity [53] and ions deficiency [54]. Results showed that the main microalgal ultrastructure changes were located in the chloroplasts and nucleus. A normal shape, unbroken membrane, ordered arrangement of grana and stroma thylakoids, and intact lamellar structure are related to the function of the chloroplast. Therefore, ultrastructural alterations in chloroplasts can cause a decline in photosynthesis [55], [56], as well as the life activities of a cell. As to the homogenous distribution of chromatin, it was observed in treated groups that chromatin formed many dense zones. Simultaneously the nuclear membrane was wrinkled. It is well known that the nucleus regulates life activities such as growth, reproduction, metabolism and protein synthesis in plant cells by genetic materials. Once the nuclear structure is damaged, the cellular physiology is disordered. Additionally, according to the pictures from the TEM, BDE-209 was more toxic to *H. akashiwo* while both its chloroplast and nucleus were clearly more badly damaged. This finding is in accordance with the result of the acute toxicity test. One possible explanation for this phenomenon is that their external structures of the cell are different. *H. akashiwo* lacks a cell wall, and therefore, the cell plasma was directly exposed to the pollutant's stress, which might have ultimately resulted in a more severe impact to the growth of this species. Though *K. mikimotoi* has no cell wall, epitheca plays an important role in ameliorating the effect of BDE-209. Collectively, the results of the acute toxicity test and the TEM data provide evidence of the potentially toxic effects of BDE-209 on marine microalgae.

The growth of both *H. akashiwo* and *K. mikimotoi* with different initial cell densities in the mono-culture was well described by the logistic equation; the Table 3 showed the summarized value of the parameters: *K*, *R^2^*, *r*, *T_P_*, *T_EP_* and *T_SP_*. Both species in the mono-cultures showed similarities at different initial cell densities: *K* value decreased with increasing initial cell densities whereas the value of *r* increased, and *T_P_*, *T_EP_* and *T_SP_* were all shortened. It was inferred that the growth of both microalgal species was density-dependent, which is similar to previous reports [57], [58], [59], [60]. One possible reason for this observation is that at different initial densities, the population utilized resources at different rates and the growth was thereby affected.

**Table 3:** Different initial biomass densities affected various parameters as well as the regression coefficients of *K. mikimotoi* and *H. akashiwo* population growth in the mono-culture as obtained by regression analysis.

	*H. akashiwo*			*K. mikimotoi*	
	0.5×10^4^cells ml^−1^	0.8×10^4^ cells ml^−1^	3.2×10^4^ cells ml^−1^	0.2×10^4^cells ml^−1^	0.5×10^4^ cells ml^−1^
*K* (×10^4^cells ml^−1^)	99.9215	90.1280	61.0542	73.6985	54.4848
*r*	0.2977	0.3675	0.5450	0.3499	0.3994
*R* ^2^	0.9709	0.9812	0.9186	0.9850	0.9604
*T*_p_ (d)	11.41	9.440	6.450	16.00	11.54
T_EP_ (d)	4	3	2	9	7
T_SP_ (d)	16	13	12	18	15

When the two species of microalgae were grown in co-culture without BDE-209 exposure, significant growth suppression occurred to either *H. akashiwo* (H) or *K. mikimotoi* (K) at initial biomass ratios of H∶K = 4∶1, 1∶1 and 1∶4 as compared to their respective mono-culture control groups (*P*<0.01). Their growth could also be described by the logistic equation, and the estimated parameters are summarized in Table 4. It was obvious to see that BDE-209 interferes with population growth for *H. akashiwo* and *K. mikimotoi* when these two species were grown in co-culture, and disparate sensitivity was one of the reasons for the different effects.

**Table 4:** Different initial biomass ratios affected parameters estimated from logistic population growth equations in the co-culture of *K. mikimotoi* (K) and *H. akashiwo* (H) with different initial biomass ratios as obtained by regression analysis.

	*K. mikimotoi*			*H. akashiwo*		
	H∶K = 4∶1	H∶K = 1∶1	H∶K = 1∶4	H∶K = 4∶1	H∶K = 1∶1	H∶K = 1∶4
*K* (×10^4^cells ml^−1^)	5.0954	11.4439	23.0079	30.6690	25.0804	—
*r* (d^−1^)	0.9715	0.3520	0.5203	0.5909	0.4810	—
*R* ^2^	0.9607	0.9673	0.9717	0.9265	0.9776	0.2395
*T*_p_ (d)	—	11.37	9.950	5.780	6.390	—
T_EP_(d)	—	9	6	2	3	—
T_SP_(d)	—	13	15	10	13	—

The interaction between *K. mikimotoi* and *H. akashiwo* was simulated by the Lotka-Volterra competition model according to the following formula [61]:
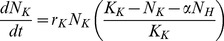
(3)

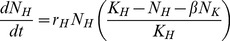
(4)In (3) and (4), *N*, *K* and *r* represent the cell density, the cell carrying capacity and the maximum growth rate for both species, respectively. Parameters *α* and *β* are nondimensional and indicate the degree of inhibition by *H. akashiwo* or *K. mikimotoi*, respectively, when compared with self-interference. The degree of inhibition can be expressed as follows:

*K*_K_>*K*_H_/*β*, *K*_H_<*K*_K_/*α*: *K. mikimotoi* out-competes *H. akashiwo*;
*K*_H_>*K*_K_/*α*, *K*_K_<*K*_H_/*β*: *H. akashiwo* out-competes *K. mikimotoi*;
*K*_K_<*K*_H_/*β*, *K*_H_<*K*_K_/*α*: the two species will co-exist and the balance is stable;
*K*_K_>*K*_H_/*β*, *K*_H_>*K*_K_/*α*: the two species will co-exist but the balance is unstable.

According to the formula of the Lotka-Volterra competition model, the value of *α* and *β* were easily calculated and then the competitive results of different initial biomass proportions were obtained with or without BDE-209. These results were similar with the one reported by Uchida [30] who found the competition between *Heterocapsa circularisquama* and *Gymnodinium mikinotoi* to be dependent upon the initial cell densities of the two species (Table 5).

**Table 5:** BDE-209 at two toxicities (l stands for low toxicity, and h stands for high toxicity) affected some parameters as well as the regression coefficients of *K. mikimotoi* (K) and *H. akashiwo* (H) population growth in the co-culture with different initial biomass ratios as obtained by regression analysis.

		*K* (×10^4^cells ml^−1^)	*r* (d^−1^)	*R* ^2^	*T*_p_ (d)	T_EP_(d)	T_SP_(d)
H∶K = 4∶1	H-l	36.5252	0.3494	0.9107	6.190	2	13
	K-l	—	—	0.7897	—	—	—
	H-h	46.0042	0.2142	0.9503	7.470	2	16
	K-h	—	—	0.7689	—	—	—
H∶K = 1∶1	H-l	28.7673	0.3964	0.9897	9.120	3	16
	K-l	13.2915	0.4598	0.9520	10.96	4	18
	H-h	29.3092	0.4247	0.9556	7.600	3	14
	K-h	12.4799	0.3233	0.9505	11.68	4	18
H∶K = 1∶4	H-l	13.2452	0.3964	0.9024	7.830	3	14
	K-l	45.2492	0.3303	0.9669	13.81	5	18
	H-h	18.6748	0.4814	0.9006	5.910	3	13
	K-h	31.4937	0.2770	0.9578	12.97	4	17

It was found that the nutrients, light density and temperature were not limiting factors during the test period; therefore, the competition between the two species revolved around space resources. A great deal of research has focused on the allelopathy between microalgae and other organisms and has proved that both *H. akashiwo* and *K. mikimotoi* can produce some bioactive materials, mainly ichthyotoxic and hemolytic compounds, to have ecological relevance as a survival strategy [62], [63]. Therefore, we can infer that allelopathic materials secreted by both species likely exist in the competition of bio-algal culture and play a vital role in the composition and succession of the microalgal community.

Many models since the 1980s have attempted to understand the response of plants to allelochemicals [28], [64], [65]. The allelopathic strength between microalgae and other organisms depends on the microalgal initial cell density, which has been demonstrated [66]. Sinkkonen [67], [68] considered that when N plants compete for a certain amount of phytochemicals, an average plant takes up one-Nth part. In the case of *H. akashiwo*, we regarded the amount of allelopathic exudates from one cell of *K. mikimotoi* as X_0_ in this study, and the total number was 0.2×10^4^ X_0_, 0.2×10^4^ X_0_ and 0.5×10^4^ X_0_ per mL in co-culture when the ratio was equal to H∶K = 4∶1, 1∶1 and 1∶4, respectively. Therefore, one cell of *H. akashiwo* suffered from 0.2×10^4^ X_0_/3.2×10^4^ (X_0_/16), 0.2×10^4^ X_0_/0.8×10^4^ (X_0_/4) and 0.5×10^4^ X_0_/0.5×10^4^ (X_0_) per mL at these three ratios, respectively. Similarly, one cell of *K. mikimotoi* separately took up 3.2×10^4^ X_1_/0.2×10^4^ (16X_1_), 0.8×10^4^ X_1_/0.2×10^4^ (4X_1_) and 0.5×10^4^ X_1_/0.5×10^4^ (X_1_) (where X_1_ stands for the amount of allelopathic materials secreted by one cell of *H. akashiwo*). Additionally, many researchers have found that allelopathic substances can stimulate growth at low concentrations but inhibit growth at high concentrations [69]. When the initial biomass ratio was equal to H∶K = 4∶1, one cell of *K. mikimotoi* was subject to suppression from 16X_1_ of the toxin, and the allelopathic strength was greater than in the groups of H∶K = 1∶1 and H∶K = 1∶4 whereas *H. akashiwo* only took up 1/16 X_0_ of the toxin and the allelopathic strength was weaker than in the other two groups. Therefore, the competitive results differed because different initial biomass causes different usage rates of resources, and the population growth rates of the two species are not same when the initial biomass ratios changed without BDE-209 exposure. Meanwhile, allelopathic compounds are concentration dependent with respect to initial cell densities.

When BDE-209 was inoculated into the co-culture medium, different groups exhibited different performances. In the co-culture with an initial biomass ratio of 4∶1, *H. akashiwo* remained to be in the dominant position. This dominance of *H. akashiwo* occurred because the growth of *K. mikimotoi* was inhibited so intensely in all groups that the growth curve could not be fit by the logistic growth model and had no obvious exponential phase. When the group of H∶K = 1∶1, the competition results changed a lot among three groups. On one hand, it was well known that the value of the coefficients stood for the inhibition between two kinds of microalgae. The α value changed much more with increasing BDE-209 concentration than that of β, which meant that the inhibiting effect of one cell on *H. akashiwo* declined largely for *K. mikimotoi*. On the other hand, *K. mikimotoi* out-competed *H. akashiwo* in both groups in the absence of the toxin and in groups with low toxicity, but the two species co-existed in groups with high toxicity as seen in a comparison of the values of *K*_K_, *K*_H_/*β*, *K*_H_ and *K*_K_/*α*. Consequently, it was found that the competition gradually shifted in favor of *H. akashiwo*, which was also demonstrated in the groups where H∶K = 1∶4. Two possible factors might be implicated in this competition. Firstly, the sensitivities of *K. mikimotoi* and *H. akashiwo* to BDE-209 were different, and therefore, the performances of these two species were dissimilar. Secondly, BDE-209 weakened the suppression from *K. mikimotoi* or heightened the allelopathic strength from *H. akashiwo*, but the mechanism needs to be further explored (Table 6).

**Table 6:** BDE-209 exposure affected parameters α and β simulated by the Lotka-Volterra competition model and the competitive results in the co-culture *K. mikimotoi* (K) and *H. akashiwo* (H) with different initial biomass ratios.

	Co-culture without BDE-209			Co-culture with low toxicity groups			Co-culture with high toxicity groups		
	α	β	result	α	β	result	α	β	result
H∶K = 4∶1	—	—	H dominates	—	—	H dominates	—	—	H dominates
H∶K = 1∶1	6.681	0.5766	K dominates	1.374	0.3013	K dominates	0.0300	0.3221	H and K coexist
H∶K = 1∶4	—	—	K dominates	0.5200	1.493	H and K coexist	0.2324	0.6628	H dominates

Currently, the actual concentration of BDE-209 was shown from <1 to 2 pg L^−1^ in the seawater of East Greenland Sea, North Sea and the Atlantic and Southern Ocean based on some reports [70], [71], [72]. However, it was reported that the high end of global BDE-209 concentrations were 335–65200 pg L^−1^ in the Pearl River Delta, China, and higher than concentrations used in this study. Hence, the microalgae may have been subjected to the negative effects brought by BDE-209 in this region. Harmful algal blooms (HABs) are a kind of marine phenomena that one or some species of phytoplankton proliferate and accumulate rapidly in the global scale that cause some negative impacts. There are many factors that may contribute to HABs, of which eutrophication plays a crucial role [73]. The large-scale initiation of HABs along the open coasts usually related to the nutrients from upwelling or advecting water masses, while the anthropogenic nutrients could be a dominate factor in estuaries, embayments or nearshore coasts where HABs originate [74]. It was also found that HABs have occurred constantly in Pearl River Delta for several years, therefore it will be meaningful to find out the relevance between persistent organic pollutions (POPs) and HABs. This study may be considered as the groundwork to provide some theoretical help.

## Conclusions

Results of the research work showed that BDE-209 did have negative impacts on the population growth and change the ultrastructures for *H. akashiwo* and *K. mikimotoi*. Moreover, BDE-209 broke the competitive balance to make competition gradually shift in favor of *H. akashiwo* based on the Lotka-Volterra competition model. We suggest that a quicker and better understanding of BDE-209 toxicity should be established which is helpful for the purpose of the reasonable use and disposal of electronics.

## References

[1] Christensen JH, Platz J (2001) Screening of polybrominated diphenyl ethers in blue mussels, marine and freshwater sediments in Denmark. J Environ Monit 3: 543–547. 11695126 10.1039/b105501c

[2] Lema SC, Schultz I, Scholz N, Incardona J, Swanson P (2007) Neural defects and cardiac arrhythmia in fish larvae following embryonic exposure to 2,2′,4,4′-tetrabromodiphenyl ether (PBDE-47). Aquat Toxicol 82: 296–307. 17412433 10.1016/j.aquatox.2007.03.002

[3] Li A, Rockne KJ, Sturchio N, Song W, Ford JC, et al (2006) Polybrominated diphenyl ethers in the sediments of the Great Lakes. 4. Influencing factors, trends, and implications. Environ Sci Technol 40: 7528–7534. 17256490 10.1021/es0609506

[4] ter Schure AFH, Larsen P, Agrell C (2004) Atmosphereic transport of polybrominated diphenyl ethers and polybrominated biphenyls to the Baltic Sea. Environ Sci Technol 38: 1282–1287. 15046327 10.1021/es0348086

[5] Law RJ, Allchin CR, de Boer J, Covaci A, Herzke D, et al (2006) Levels and trends of brominated flame retardants in the European environment. Chemosphere 64: 187–208. 16434081 10.1016/j.chemosphere.2005.12.007

[6] USEPA (United States Environmental Protection Agency) (2010) An exposure assessment of polybrominated diphenyl ethers. Washington, DC: USEPA; p. 378.

[7] Asplund L, Hornung M, Peterson RE, Turesson K, Bergman A (1999) Levels of polybrominated diphenyl ethers (PBDEs) in fish from the Great Lakes and Baltic Sea. Organohalogen Compd 40: 351–354.

[8] Covaci A, Losada S, Roosens L, Vetter W, Santos FJ, et al (2008) Anthropogenic and naturally occurring organobrominated Compounds in two deep-Sea fish species from the Mediterranean Sea. Environ Sci Technol 42: 8654–8660. 19192777 10.1021/es8016528

[9] Christensen JR, MacDuffee M, Macdonald RW, Whiticar M, Ross PS (2005) Persistent organic pollutants in British Columbia grizzly bears: consequence of divergent diets. Environ Sci Technol 39: 6952–6960. 16201616 10.1021/es050749f

[10] Van den Steen E, Covaci A, Jaspers VLB, Dauwe T, Voorspoels S, et al (2007) Accumulation, tissue-specific distribution and debromination of decabromodiphenyl ether (BDE 209) in European starlings (*Sturnus vulgaris*). Environ Pollut 148: 648–653. 17239511 10.1016/j.envpol.2006.11.017

[11] Johnson-Restrepo B, Kannan K, Addink R, Adams DH (2005) Polybrominated diphenyl ethers and polychlorinated biphenyls in a marine foodweb of coastal Florida. Environ Sci Technol 39: 8243–8250. 16294860 10.1021/es051551y

[12] Zhou T, Taylor MM, DeVito MJ, Crofton KM (2002) Developmental exposure to brominated diphenyl ethers results in thyroid hormone disruption. Toxicol Sci 66: 105–116. 11861977 10.1093/toxsci/66.1.105

[13] Darnerud PO, Eriksen G, Johannesson T, Larsen P, Viluksela M (2001) Polybrominated diphenyl ethers: occurrence, dietary exposure, and toxicology. Environ Health Perspect 109: 49–68. 11250805 10.1289/ehp.01109s149 PMC1240542

[14] Kuriyama SN, Talsness CE, Grote K, Chahoud I (2005) Developmental Exposure to Low-Dose PBDE-99: Effects on Male Fertility and Neurobehavior in Rat Offspring. Environ Health Perspect 113: 149–154. 15687051 10.1289/ehp.7421 PMC1277857

[15] Van der Ven LTM, van de Kuil T, Leonards PEG, Slob W, Canton RF, et al (2008) A 28-day oral dose toxicity study in Wistar rats enhanced to detect endocrine effects of decabromodiphenyl ether (deca-BDE). Toxicol Lett 179: 6–14. 18495385 10.1016/j.toxlet.2008.03.003

[16] Leberf M, Couillard CM, Legare B (2006) Effects of DeBDE and PCB-126 on hepatic concentrations of PBDEs and methoxy-PBDEs in Atlantic tomcod. Environ Sci Technol 40: 3211–3216. 16749683 10.1021/es0521829

[17] European Commission (2004) Update of the Risk Assessment of Bis(pentabromophenyl) ether (decabromodiphenyl ether). Final Environmental Draft of May 2004, R013_0405_env. Available: http://ec.europa.eu/health/ph_risk/committees/04_scher/docs/scher_o_012.pdf

[18] European Commission (2003) Diphenyl ether, Octabromo dericative. Summary risk assessment report. Available: http://www.pic.int/Portals/5/download.aspx?d=UNEP -FAO-RC-CRC.7-10- Add.2-4.En.pdf

[19] Källqvist T, Grung M, Tollefsen KE (2006) Chronic toxicity of 2,4,29,49-tetrabromodiphenyl ether on the marine alga *Skeletonema Costatum* and the crustacean *Daphnia magna* . Environ Toxicol Chem 25: 1657–1662. 16764486 10.1897/05-424r.1

[20] Meng FP, Li ZN, Zhao SS, Liu J (2009) Effects of BDE-47 on the antioxidase activities of four species of marine microalgae. Eco Environ Sci 18: 1659–1664.

[21] Zhang XX, Tang XX, Jiang S, Yuan MQ, Zhou B (2012) Toxic effect of 2,2′,4,4′- Tetrabromodiphenyl Ether (BDE-47) on *Karenia mikimotoi* in the different levels of biological organizations. Mar Environ Sci (accepted)..

[22] Sommer U, Maciej GZ, Lampert W (1986) The PEG-model of seasonal succession of planktonic events in fresh waters. Fundam Appl Toxicol 106: 433–471.

[23] Litchman E, Klausmeier CA (2001) Competition of phytoplankton under fluctuating light. Am Nat 157: 170–187. 18707270 10.1086/318628

[24] Mulderij G, Mooij WM, Van Donk E (2005) Allelopathic growth inhibition and colony formation of the green alga *Scenedesmus obliquus* by the aquatic macrophytes *Stratiotes aloides* . Aquat Ecol 39: 11–21.

[25] Gross EM (2003) Allelopathy of aquatic autotrophs. Crit Rev Plant Sci 22: 313–339.

[26] Roy S, Alam S, Chattopadhyay J (2006) Competing effects of toxin-producing phytoplankton on overall plankton populations in the Bay of Bengal. Bull Math Biol 68: 2303–2320. 16804650 10.1007/s11538-006-9109-5

[27] Nakamaru M, Iwasa Y (2000) Competition by allelopathy proceeds in traveling waves: colicin -immune strain aids colicin-sensitive strain. Theor Popul Biol 57: 131–144. 10792978 10.1006/tpbi.1999.1448

[28] Wang Y, Tang XX (2008) Interactions between *Prorocentrum donghaiense* Lu and *Scrippsiella trochoidea* (Stein) Loeblich III under laboratory culture. Harmful Algae 7: 65–75.

[29] Iwasa Y (Ed.) (1998) Suri-seibutugaku nyuumon. 2nd ed. KyorituSyuppan, Tokyo, p. 352.

[30] Uchida T, Toda S, Matsuyama Y, Yamaguchi M, Kotani Y, et al (1999) Interactions between the red tide dinoflagellates *Heterocapsa circularisquama* and *Gymnodinium mikinotoi* in laboratory culture. J Exp Mar Biol Ecol 241: 285–299.

[31] Solé J, García-Ladona E, Ruardij P, Estrada M (2005) Modelling allelopathy among marine algae. Ecol Model 183: 373–384.

[32] Chattopadhyay J (1996) Effect of toxic substances on a two-species competitive system. Ecol Model 84: 287–289.

[33] Goodman JE (2009) Neurodevelopmental effects of decabromodiphenyl ether (BDE-209) and implications for the reference dose. Regul Toxicol Pharmacol 54: 91–104. 19249332 10.1016/j.yrtph.2009.02.006

[34] BSEF (2003) Major Brominated Flame Retardants Volume Estimates, http://www.bsef-site.com/docs/bfr_vols_2001.doc.

[35] Schecter A, Päpke O, Harris TR, Tung KC (2006) Partitioning of polybrominated diphenyl ether (PBDE) congeners in human blood and milk. Toxicol Environ Chem 88: 319–324.

[36] Madhu NV, Reny PD, Meenu Paul, Ullas N, Resmi P (2011) Occurrence of red tide caused by *Karenia mikimotoi* (toxic dinoflagellate) in the Southwest coast of India. Indian J Mar Sci 40: 821–825.

[37] Imai I, Itakura S, Yamaguchi M, Honjo T (1996) Selective germination of *Heterosigma akashiwo* (Raphidophyceae) cysts in bottom sediments under low light conditions: a possible mechanism of redtide initiation. In: Yasumoto, T., Oshima, Y. & Fukuyo, Y. (ed.), Harmful and toxic algal blooms. UNESCO, pp. 197–200.

[38] Oris JT, Giesy JP Jr (1985) The photo-enhanced toxicity of anthracene to juvenile sunfish (*Lepomis spp*). Aquat Toxicol 6: 133–146.

[39] Guillard RRL (1975) Culture of phytoplankton for feeding marine invertebrates. In: Smith, W.L., Chanley, M.H. (Eds.), Culture of Marine Animals. Plenum Press, New York, pp. 26–60.

[40] Jones MN (1984) Nitrate reduction by shaking with cadmium, alternative to cadmium columns. Water Res 18: 643–646.

[41] Hager SW, Gordon LI, Park PK (1968) A practical manual for the use of Technicon Autoanalyzer in seawater nutrient analysis. A final report To B.C.F., Contract 14-17-0001-1759.

[42] SIS (1991) Determination of acute lethal toxicity of chemical substances and effluents to *Nitocra spinipes* Boeck–Static procedure (in Swedish) Swedish Standard SS 02 81 06. SIS–Standardiseringskommissionen i Sverige, Stockholm, Sweden, pp. 17.

[43] APHA (1985) Standard methods for examination of water and wastewater. Washington DC. Sixteenth edition.

[44] Moreno-Garrido I, Lubián LM, Soares AMVM (2000) Influence of cellular density on determination of EC_50_ in microalgal growth inhibition tests. Ecotoxicol Environ Safe 47: 112–116. 10.1006/eesa.2000.1953 11023688

[45] Cavender-Bares KK, Rinaldo A, Chisholm SW (2001) Microbial size spectra from natural and nutrient enriched ecosystems. Limno Oceanogr 46: 778–789.

[46] Zhu LX, Cheng NQ, Gao X (1983) Electron microscopy in biology. Peking University Press, Beijing, China. p. 38–62.

[47] Birnbaum LS, Staskal DF (2004) Brominated flame retardants: cause for concern? Environ Health Perspect 112: 9–17. 14698924 10.1289/ehp.6559 PMC1241790

[48] Hardy M (2000) Distribution of decabromodiphenyl oxide in the environment. Organohalogen Compds 47: 237–240.

[49] Sellström U, Kierkegaard A, Alsberg T, Jonsson P, Wahlberg C, et al (1999) Brominated flame retardants in sediments from European estuaries, the Baltic sea and in sewage sludge. Organohalogen Compds 40: 383–386.

[50] WHO (1994) Brominated Diphenyl Ethers. WHO IPCS Environmental Health Criteria Document, 162 Geneva, World Health Organization.

[51] Burreau S, Zebühr Y, Broman D, Ishaq R (2004) Biomagnification of polychlorinated biphenyls (PCBs) and polybrominated diphenyl ethers (PBDEs) studied in pike (*Esox lucius*), perch (*Perca fluviatilis*) and roach (*Rutilus rutilus*) from the Baltic Sea. Chemosphere 55: 1043–1052. 15051373 10.1016/j.chemosphere.2003.12.018

[52] Peng Q, Zhou Q (2009) Influence of lanthanum on chloroplast ultrastructure of soybean leaves under ultraviolet-B stress. J Rare Earth 27: 304–307.

[53] Gabara B, Sklodowska M, Wyrwicka A, Glinska S, Gapinska M (2003) Changes in the ultrastructure of chloroplasts and mitochondria and antioxidant enzyme activity in *Lycopersicon esculentum* Mill. Leaves sprayed with acid rain. Plant Sci 164: 507–516.

[54] Yamane K, Rahman MS, Kawasaki M, Taniguchi M, Miyake H (2004) Pretreatment with a low concentration of methyl viologen decreases the effects of salt stress on chloroplast ultrastructure in rice leaves (*Oryza sativa* L.). Plant Prod Sci 7: 435–441.

[55] Bondada BR, Sams CE, Deyton DE, Cummins JC (1998) Apple and peach shoot surface morphology and soybean oil retention as influenced by simulated rainfall and soybean oil emulsions. J Am Soc Hort Sci 125: 553–557.

[56] Zhang MP, Zhang CJ, Yu GH, Jiang YZ, Strasser RJ, et al (2010) Changes in chloroplast ultrastructure, fatty acid component of thylakoid membrane and chlorophyll a fluorescence transient in flag leaves of a super-high-yield hybrid rice and its parents during the reproductive stage. J Plant Physiol 167: 277–285. 20004497 10.1016/j.jplph.2009.09.017

[57] Dong YW, Dong SL, Liu XY (2004) The effect of initial cell density on the population competition between *Alexandrium tamarense* Balech and *Heterosigma akashiwo* Hada. J Ocean Univ China 34: 964–968.

[58] Cai HJ, Tang XX, Zhang PY (2005) Effects of initial cell density on the population growth of three species of red tide microalgae. Mar Evrion Sci 24: 37–39.

[59] Xie ZH, Xiao H, Cai HJ, Tang XX (2008) The effect of initial cell density on the interspecific competition between *Heterosigma akashiwo* and *Prorocentrum donghaiense* . Mar Evrion Sci 27: 462–465.

[60] Zhao XW, Tang XX, Wang Y (2009) Interactions between two species of marine bloom microalgae under controlled laboratory conditions: *Heterosigma akashiwo* and *Karenia mikimotoi* . Chinese J Plant Ecol 33: 958–965.

[61] Lampert W, Sommer U (2007) Limnoecology. Oxford University Press, New York, USA. p. 89–147.

[62] Twiner MJ, Chidiac P, Dixon SJ, Trick CG (2005) Extracellular organic compounds from the ichthyotoxic red tide alga *Heterosigma akashiwo* elevate cytosolic calcium and induce apoptosis in Sf9 cells. Harmful Algae 4: 789–800.

[63] Zou Y, Yamasaki Y, Matsuyama Y, Yamaguchi K, Honjo T, et al (2010) Possible involvement of hemolytic activity in the contact-dependent lethal effects of the dinoflagellate *Karenia mikimotoi* on the rotifer *Brachionus plicatilis* . Harmful Algae 9: 367–373.

[64] Carballeira A, Carral E, Reigosa MJ (1988) Asymmetric Small-Scale Distribution and Allelopathy: Interaction between *Rumex obtusifolius* L. and Meadow Species. J Chem Ecol 14: 1775–1786. 24276533 10.1007/BF01014643

[65] Weidenhamer JD, Hartnett DC, Romeo JT (1989) Density-dependent phytotoxicity: Distinguishing resource competition and allelopathic interference in plants. J AppL Ecol 26: 613–624.

[66] Sinkkonen A (2005) Modeling the effect of density-dependent chemical interference upon seed germination. Nonlinearity Biol Toxicol Med 3: 225–233. 19330163 10.2201/nonlin.003.02.004 PMC2657952

[67] Sinkkonen A (2001) Density-dependent chemical interference—an extension of the biological response model. J Chem Ecol 27: 1513–1523. 11504041 10.1023/a:1010329612753

[68] Sinkkonen A (2003) A model describing chemical interference caused by decomposing residues at different densities of growing plants. Plant Soil 250: 315–322.

[69] Wang Y, Yu Z, Song XX, Zhang SD (2006) Interactions between the bloom forming dinoflagellates *Prorocentrum donghaiense* and *Alexandrium tamarense* in laboratory culture. J Sea Res 56: 17–26.

[70] Möller A, Xie Z, Sturm R, Ebinghaus R (2011) Polybrominated diphenyl ethers (PBDEs) and alternative brominated flame retardants in air and seawater of the European Arctic. Environ Pollut 159: 1577–1583. 21421283 10.1016/j.envpol.2011.02.054

[71] Xie ZY, Möller A, Ahrens L, Sturm R, Ebinghaus R (2011) Brominated flame retardants in seawater and atmosphere of the Atlantic and the Southern Ocean. Environ Sci Technol 45: 1820–1826. 21291232 10.1021/es103803t

[72] Möller A, Xie ZY, Caba A, Sturm R, Ebinghaus R (2012) Occurrence and air-seawater exchange of brominated flame retardants and Dechlorane Plus in the North Sea. Atmos Environ 46: 346–353.

[73] Sellner KG, Doucette GJ, Kirkpatrick GJ (2003) Harmful algal blooms: causes, impacts and detection. J Ind Microbiol Biotechnol 30: 383–406. 12898390 10.1007/s10295-003-0074-9

[74] Anderson DM, Burkholder JM, Cochlan WP, Glibert PM, Gobler CJ, et al (2008) Harmful algal blooms and eutrophication: Examining linkages from selected coastal regions of the United States. Harmful Algae 8: 39–53. 19956363 10.1016/j.hal.2008.08.017 PMC2677713

